# The Role of Neutrophil Proteins on the Amyloid Beta-RAGE Axis

**DOI:** 10.1371/journal.pone.0163330

**Published:** 2016-09-27

**Authors:** Amanda J. Stock, Anne Kasus-Jacobi, Jonathan D. Wren, Virginie H. Sjoelund, Glenn D. Prestwich, H. Anne Pereira

**Affiliations:** 1 Oklahoma Center for Neuroscience, University of Oklahoma Health Sciences Center, Oklahoma City, Oklahoma, United States of America; 2 Department of Pharmaceutical Sciences, University of Oklahoma Health Sciences Center, Oklahoma City, Oklahoma, United States of America; 3 Arthritis and Clinical Immunology Research Program, Oklahoma Medical Research Foundation, Oklahoma City, Oklahoma, United States of America; 4 Department of Biochemistry and Molecular Biology, University of Oklahoma Health Sciences Center, Oklahoma City, Oklahoma, United States of America; 5 Core Laboratory for Molecular Biology and Cytometry Research, University of Oklahoma Health Sciences Center, Oklahoma City, Oklahoma, United States of America; 6 Department of Medicinal Chemistry, University of Utah, Salt Lake City, Utah, United States of America; 7 Department of Pathology, University of Oklahoma Health Sciences Center, Oklahoma City, Oklahoma, United States of America; 8 Department of Cell Biology, University of Oklahoma Health Sciences Center, Oklahoma City, Oklahoma, United States of America; Rutgers University, UNITED STATES

## Abstract

We previously showed an elevated expression of the neutrophil protein, cationic antimicrobial protein of 37kDa (CAP37), in brains of patients with Alzheimer’s disease (AD), suggesting that CAP37 could be involved in AD pathogenesis. The first step in determining how CAP37 might contribute to AD pathogenesis was to identify the receptor through which it induces cell responses. To identify a putative receptor, we performed GAMMA analysis to determine genes that positively correlated with CAP37 in terms of expression. Positive correlations with ligands for the receptor for advanced glycation end products (RAGE) were observed. Additionally, CAP37 expression positively correlated with two other neutrophil proteins, neutrophil elastase and cathepsin G. Enzyme-linked immunosorbent assays (ELISAs) demonstrated an interaction between CAP37, neutrophil elastase, and cathepsin G with RAGE. Amyloid beta 1–42 (Aβ_1–42_), a known RAGE ligand, accumulates in AD brains and interacts with RAGE, contributing to Aβ_1–42_ neurotoxicity. We questioned whether the binding of CAP37, neutrophil elastase and/or cathepsin G to RAGE could interfere with Aβ_1–42_ binding to RAGE. Using ELISAs, we determined that CAP37 and neutrophil elastase inhibited binding of Aβ_1–42_ to RAGE, and this effect was reversed by protease inhibitors in the case of neutrophil elastase. Since neutrophil elastase and cathepsin G have enzymatic activity, mass spectrometry was performed to determine the proteolytic activity of all three neutrophil proteins on Aβ_1–42_. All three neutrophil proteins bound to Aβ_1–42_ with different affinities and cleaved Aβ_1–42_ with different kinetics and substrate specificities. We posit that these neutrophil proteins could modulate neurotoxicity in AD by cleaving Aβ_1–42_ and influencing the Aβ1–42 –RAGE interaction. Further studies will be required to determine the biological significance of these effects and their relevance in neurodegenerative diseases such as AD. Our findings identify a novel area of study that underscores the importance of neutrophils and neutrophil proteins in neuroinflammatory diseases such as AD.

## Introduction

Neutrophil proteins are essential components of the innate immune system, and contribute to host defense by stimulating cytokine production, destroying invading pathogens, and recruiting other immune cells to sites of infection and inflammation [1–4]. Although the brain is considered an immune privileged site where minimal inflammatory responses can be elicited [5, 6] a number of immune mediators including neutrophil proteins have been detected in the brain parenchyma. Studies have shown increased levels of neutrophil proteins such as myeloperoxidase [7] and α-defensins 1 and 2 [8] in patients with neuroinflammatory diseases, including Alzheimer’s disease (AD). Our lab previously observed the increased expression of the neutrophil cationic antimicrobial protein of 37kDa (CAP37) in cerebrovascular endothelial cells in the hippocampus of AD patients [9]. In a more recent study, we demonstrated the upregulation of CAP37 expression in cortical pyramidal neurons of AD patients [10]. We also observed cerebral expression of neutrophil elastase and cathepsin G, two other neutrophil proteins with sequence homology to CAP37. Increased expression of CAP37 was found in the brains of patients with AD compared with normal age matched controls, whereas levels of neutrophil elastase and cathepsin G were not elevated in AD patients [10]. These observations led to our hypothesis that CAP37 was a likely player in the neuroinflammatory process underlying AD.

One way that CAP37 and other neutrophil proteins could mediate neuroinflammation is by activating inflammatory receptors. Microglia are the predominant cells that regulate inflammatory responses in the brain. A previous report from our lab demonstrated that CAP37 was a potent modulator of microglial functions [2], indicating that a receptor for CAP37 may exist on microglial cells. Much is still unknown regarding the specific mechanisms of cell responses induced by CAP37-receptor-mediated interactions, and the identity of the CAP37 receptor(s) in the brain remains elusive. By performing a gene correlation analysis called GAMMA [11], we could determine genes that positively correlated with CAP37 and obtain clues for potential CAP37 receptors. Results obtained from GAMMA analysis prompted us to investigate interactions between CAP37 and the receptor for advanced glycation end products (RAGE).

RAGE is an inflammatory receptor expressed on various brain cells, including microglia, endothelial cells, astrocytes, and neurons [12]. RAGE expression is high in neurons during development, but expression is low in brain cells of adults during normal physiological conditions [12]. A number of ligands for RAGE have been identified, including advanced glycation end products (AGEs), which are well known for their role in diabetes and athlerosclerosis, inflammatory mediators such as members of the S100/calgranulin family, high mobility group box 1 protein (HMGB-1), the Mac-1 integrin, and amyloid beta (Aβ), found in the senile plaques of AD brains [13–15]. RAGE activation by its ligands initiates a positive feedback loop of inflammation by inducing de novo synthesis of NF-κBp65 mRNA and protein, and in this way contributes to chronic production of pro-inflammatory cytokines, up-regulation of RAGE, and inflammation [13, 16]. This chronic inflammatory response has been reported to occur in many neuroinflammatory diseases including AD. Furthermore, RAGE expression is increased in the brains of patients with AD [17], allowing for increased Aβ-RAGE signaling.

The general consensus is that Aβ is a major factor augmenting the neurotoxicity and cognitive decline observed in patients with AD [18]. The two most prevalent forms of Aβ are amyloid beta 1–40 (Aβ_1–40_) and amyloid beta 1–42 (Aβ_1–42_). Aβ_1–42_ is the most toxic species that accumulates in the brains of AD patients [19]. RAGE activation by Aβ_1–42_ induces oxidative stress in cerebral endothelial cells and astrocytes by stimulating NADPH oxidase to produce reactive oxygen species (ROS) [20, 21]. The Aβ-RAGE interaction also induces NFκB activation on neurons and microglia which contributes to a pro-inflammatory environment [22, 23].

In the current study, we investigated binding of CAP37 to RAGE, and measured binding of neutrophil elastase and cathepsin G to RAGE for comparison. Additionally, we investigated the effects of CAP37, neutrophil elastase, and cathepsin G on the interaction of RAGE with amyloid beta (Aβ). Disruption of the Aβ_1–42_ -RAGE interaction could be neuroprotective by decreasing oxidative stress and inflammation in the AD brain. On the other hand, enhancing this interaction could accelerate neurotoxicity. Understanding the effects of neutrophil proteins on the Aβ_1–42_ -RAGE axis could be important for developing neuroprotective AD therapeutics.

## Materials and Methods

### Materials

Recombinant human RAGE Fc chimera was purchased from R&D Systems Inc. (Minneapolis, MN). CAP37, cathepsin G, and neutrophil elastase purified from human neutrophils were purchased from Athens Research & Technology (Athens, GA). All neutrophil proteins were determined by the manufacturer to be >95% pure by SDS-PAGE. For purified CAP37, chromogenic activity assays using synthetic substrates specific for either neutrophil elastase or cathepsin G were performed by the manufacturer to rule out the presence of neutrophil elastase and cathepsin G. Activity assays were also performed to confirm the absence of cathepsin G in purified neutrophil elastase and vice versa. Essentially fatty acid free bovine serum albumin (BSA, cat# A3803) was from Sigma Aldrich (St. Louis, MO). Amyloid beta (Aβ_1–42_) purchased from American Peptide Company (Sunnyvale, CA) and Bachem (Torrance, CA) was determined to be 95.6% and 95.2% pure by HPLC analysis, respectively. The RAGE antagonist used was a new class of sulfated anionic polysaccharide semi-synthetic glycosaminoglycan ethers known as GM-0111 [24]. Protease inhibitor cocktail tablets were purchased from Roche Life Science (Indianapolis, IN) and used at 1X concentration. The primary antibodies used were monoclonal mouse anti-amyloid precursor protein/amyloid beta (APP/Aβ, #2450, Cell Signaling, Danvers, MA), polyclonal goat anti-RAGE (#AF1145, R&D Systems), and polyclonal rabbit anti-RAGE (#ab37647, Abcam, Cambridge, MA). Rabbit anti-goat secondary antibody conjugated to horseradish peroxidase (HRP) was purchased from Pierce (Rockford, IL). Donkey anti-mouse and donkey anti-rabbit secondary antibodies conjugated to HRP were purchased from Jackson ImmunoResearch Laboratories (West Grove, PA).

### GAMMA

Global Microarray Meta-Analysis (GAMMA) [11] was used to identify genes that positively correlated with CAP37 (gene expressions are increased or decreased concomitantly) across heterogeneous datasets. GAMMA used the entire curated set (GDS files) of 3,900 2-color human microarray datasets downloaded from NCBI’s GEO database [25] and normalized. The 20 most correlated genes were then analyzed for established shared protein-protein interactions (PPIs) within the Human Protein Reference Database (HPRD) [26].

### ELISAs

Aβ_1–42_ used for ELISAS was purchased from Bachem, dissolved in 1 mM sodium hydroxide, aliquoted, and kept at -20°C until used. Nunc Maxisorp 96 well plates (VWR, Radnor, PA) were coated with 100 μL of phosphate buffered saline (PBS, pH 7.4) containing the stated concentrations of CAP37, neutrophil elastase, cathepsin G, Aβ_1–42_, RAGE, or BSA for 3 h at room temperature and then overnight at 4°C. The plates were washed three times with PBS containing 0.05% tween (PBST), and then blocked in PBST containing 3% bovine serum albumin (BSA). Indicated proteins were added to the plate in 50 μL PBST containing 0.1% BSA and incubated at 37°C for 70 mins. Wells were washed four times with PBST, and then incubated with 50 μL of goat anti-RAGE primary antibody (0.5 μg/ml) or mouse anti-APP/Aβ antibody (0.5 μg/ml-1 μg/ml) for 1 h. After washing the wells four times with PBST, 50 μL of rabbit anti-goat at 0.16 μg/ml or donkey anti-mouse at 0.04–0.08 μg/ml secondary antibodies were added for 1 h at room temperature. Wells were washed with PBST before using o-phenylenediamine (OPD) substrate (Sigma-Aldrich) to develop a colorimetric reaction based on the amount of bound RAGE or Aβ_1–42_. Plates were incubated with OPD substrate for 10–30 minutes in the dark before adding 2.5M H_2_SO_4_ to stop the reaction, and a Synergy 2 multi-detection microplate reader (Biotek Instrument Inc.,Winooski, VT) was used to measure absorbance at 492 nm.

#### Binding of RAGE to neutrophil proteins

In one set of experiments aimed to determine the binding of RAGE to CAP37, neutrophil elastase, and cathepsin G, maxisorp plates were coated with CAP37 (0.5 μg/well, 208 nM), neutrophil elastase (0.5 μg/well, 192 nM), cathepsin G (0.5 μg/well, 200 nM), Aβ_1–42_ (0.5 μg/well, 1 μM), or BSA (1000 μg/well, 152 μM) overnight at 4°C. Recombinant human RAGE Fc chimera (0–0.01 μg/well, 0–3.3 nM) that pre-incubated in the presence or absence of an excess of RAGE antagonist, GM-0111 (0.25 μg/ml, 50 nM) with rotation overnight at 4°C, was added to wells of the coated plate. Goat anti-RAGE primary antibody at 0.5 μg/ml was used to detect bound RAGE.

#### Competitive inhibition of Aβ_1–42_ binding to RAGE by neutrophil proteins

In another set of experiments aimed to determine if the neutrophil proteins could competitively inhibit the binding of Aβ_1–42_ to RAGE, the maxisorp plates were coated with RAGE Fc chimera (0.5 μg/well, 83.3 nM) overnight at 4°C. The next day Aβ_1–42_ (0.0025 μg/well, 11 nM) ± a protease inhibitor cocktail was added to respective wells at the same time as varying concentrations of CAP37 (0–3 μg/well, 0–2.5 μM) or neutrophil elastase (0–0.65 μg/well, 0–0.5 μM).

#### Determine if neutrophil proteins interact with Aβ_1–42_ to prevent it from binding to RAGE

Another set of ELISA experiments were performed to determine if the amyloid beta-RAGE interaction could be inhibited by pre-incubation of Aβ_1–42_ with neutrophil proteins. In this set of experiments, the maxisorp plates were coated with RAGE Fc chimera (0.5 μg/well, 83.3 nM) overnight at 4°C. Aβ_1–42_ (0–0.05 μg/well, 0–222 nM) was pre-incubated with rotation overnight at 4°C in the presence or absence of CAP37 (0.025–0.1 μg/well, 21–83 nM) or neutrophil elastase (0.025–0.1 μg/well, 20–80 nM) and added to the coated plate. Mouse anti-APP/Aβ (1 μg/ml) was used to detect bound Aβ_1–42_.

#### Binding of Aβ_1–42_ to neutrophil proteins

In a final set of ELISA experiments aimed to determine the binding of Aβ_1–42_ to CAP37, neutrophil elastase, and cathepsin G, maxisorp plates were coated with CAP37 (0.5 μg/well, 208 nM), neutrophil elastase (0.5 μg/well, 192 nM), cathepsin G (0.5 μg/well, 200 nM), or BSA (1000 μg/well, 152 μM). The next day varying concentrations of Aβ_1–42_ (0–0.025 μg/well, 0–111 nM) in PBST buffer containing 0.1% BSA and a protease inhibitor cocktail was added to determine the binding of Aβ_1–42_ to CAP37, neutrophil elastase, or cathepsin G. Mouse anti-APP/Aβ at 0.5 μg/ml was used to detect bound Aβ_1–42_.

### Far-Western dot Blotting

Aβ_1–42_ used for far-Western dot blotting was purchased from Bachem, dissolved in 1 mM sodium hydroxide, aliquoted, and stored at -20°C until used. The stated concentrations of CAP37, neutrophil elastase, cathespin G, Aβ_1–42_, or BSA were each combined with 150 μL PBS and spotted through wells of a BIO-DOT apparatus (Bio-Rad, Hercules, CA) onto nitrocellulose membranes pre-soaked in PBS. After spotting, membranes were rinsed with ultrapure water, and Ponceau S (Sigma Aldrich) was used to stain for spotted proteins. Ponceau S was rinsed off with ultrapure water, and membrane blots were blocked for 2 h at room temperature in PBST containing 3% BSA. Blots were incubated with the indicated proteins (RAGE or Aβ_1–42_) diluted in PBST containing 0.1% BSA overnight with rocking at 4°C. Blots were washed three times with PBST for 10 min, and then incubated with either rabbit anti-RAGE primary antibody (0.8μg/ml) or mouse anti-APP/Aβ (0.25μg/ml) for 2 h at room temperature. Blots were washed three times with PBST for 10 min, and then incubated with donkey anti-rabbit or donkey anti-mouse secondary antibodies at 0.04μg/ml for 45 min at room temperature. Blots were washed three times with PBST. Enhanced chemiluminescent or SuperSignal West femto maximum sensitivity substrates (Pierce) were used to develop blots. Mean dot densities were quantified and normalized to ponceau S dot densities using Image J software (National Institutes of Health (NIH), Bethesda, MD).

#### Binding of RAGE to neutrophil proteins

To determine binding of RAGE to neutrophil proteins using far-Western dot blot analysis, 0.5 μg Aβ_1–42_ (as a positive control) and 1 μg of CAP37, neutrophil elastase, cathepsin G, and BSA were each spotted in quadruplicate using a BIO-DOT apparatus onto nitrocellulose membranes pre-soaked in PBS. Membranes were cut to make two blots (one for RAGE and one for RAGE+GM-0111) that each contained duplicate spots for each protein. After blocking, blots were incubated overnight at 4°C with recombinant human RAGE Fc chimera (0.25μg/ml, 4.2nM) that pre-incubated in the presence or absence of RAGE antagonist GM-0111 (0.625μg/ml, 125nM) with rotation overnight at 4°C. Rabbit anti-RAGE primary antibody at 0.8 μg/ml was used to detect bound RAGE.

#### Binding of Aβ_1–42_ to neutrophil proteins

To determine binding of Aβ_1–42_ to neutrophil proteins using far-Western dot blot analysis, 1 μg of CAP37, neutrophil elastase, cathepsin G, and BSA were each spotted in triplicate using a BIO-DOT apparatus onto nitrocellulose membranes pre-soaked in PBS. After blocking, Aβ_1–42_ was added at 0.25μg/ml (56nM) in PBST containing 0.1% BSA and a protease inhibitor cocktail. Blots were incubated with rocking overnight at 4°C. Mouse anti-APP/Aβ at 0.25μg/ml was used to detect bound Aβ_1–42_.

### Mass Spectrometry

Aβ_1–42_ used for mass spectrometry was purchased from American Peptide Company, dissolved in 0.05M Tris buffer, pH 8.0, aliquoted, and kept at -20°C until used. Samples (Aβ_1–42_ alone, or Aβ_1–42_ incubated with CAP37, neutrophil elastase, or cathepsin G) were analyzed by matrix-assisted laser-desorption/ionization mass spectrometry (MALDI-TOF MS). Concentrations of Aβ_1–42_, CAP37, neutrophil elastase, and cathepsin G used were ~75 μM, 28 μM, 7 μM, and 7 μM, respectively. All samples were incubated ± protease inhibitor cocktail. The protease inhibitor cocktail and each protein by itself were analyzed to distinguish between peaks derived from the protease inhibitors or neutrophil proteins and the peaks generated from Aβ cleavage. All samples incubated in a 1:1 volume ratio of Tris buffer pH 8.0 (containing Aβ_1–42_ or not) and sodium buffer, pH 5.5 (containing neutrophil protein or not). The sodium buffer contained 50 mM Na acetate and 150 mM NaCl. Samples were analyzed immediately after combining the neutrophil proteins with Aβ_1–42_ at room temperature (t≈ 0.25 min), after incubation at room temperature with CAP37 for 1–300 min, or after incubation at room temperature with neutrophil elastase or cathepsin G for 1–60 min. Saturated sinapinic acid was dissolved in TA30 solvent (70:30 volume ratio of 0.1% trifluoroacetic acid: acetonitrile) at 10mg/ml and was deposited onto a MTP 384-spot ground steel target plate TF, to serve as matrix and allowed to dry. Following this, the samples were combined at a 1:1 ratio with sinapinic acid solution, deposited onto respective matrix spots on the target plate, and allowed to dry. The samples were analyzed with a Bruker Ultraflex II TOF/TOF mass spectrometer (Ultraflex II, Bruker, Billerica, MA) in linear positive (LP) mode to detect a mass range of 3,000 to 30,000 daltons and reflectron positive (RP) mode to detect a mass range of 500 to 5,000 daltons. The same samples analyzed by MS were also analyzed by tandem mass spectrometry (MS/MS) using the matrix assisted laser-desorption/ionization time-of-flight/time-of-flight (MALDI-TOF/TOF) technique in collision induced dissociation mode. FlexControl software (Bruker) was used to operate the spectrometer and flexAnalysis software (Bruker) was used to analyze and process the spectra.

### Statistical Analysis

All statistics were performed using Graph Pad Prism 6 (GraphPad Software, La Jolla, CA). Data are represented as mean± SEM of results. To analyze RAGE and Aβ_1–42_ binding to each of the neutrophil proteins with ELISAs, the optical density (OD) values were log transformed. Two-way analysis of variances (ANOVAs) were performed with Dunnett’s multiple comparison tests. Student’s unpaired *t* tests were performed to compare RAGE binding in the presence or absence of the RAGE antagonist, GM-0111 (values not in log). Kruskal-Wallis tests with Dunn’s multiple comparison tests were used to analyze binding by Far-Western dot blotting (see figure legends 3 and 6 for details on multiple comparison tests). For analyzing Aβ_1–42_ binding to RAGE with ELISAs, Kruskal Wallis tests were performed with Dunn’s multiple comparisons test (see figure legends 4 and 5 for details on multiple comparison tests). To obtain dissociation constants (*K*_*d*_), half-maximal inhibitory concentrations (IC_50_), and maximum absorbance values, BSA was subtracted as background and values were fitted to a curve of nonlinear regression.

## Results

### CAP37 expression is highly correlated with ligands of RAGE

**Fig 1:**
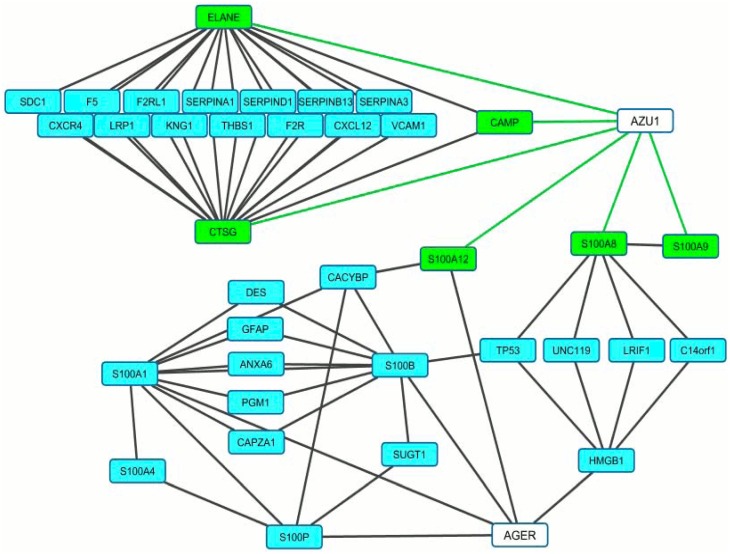
CAP37 expression is highly correlated with ligands of RAGE. Figure shows a network of genes correlated with *AZU1* (CAP37) and how they are associated with *AGER* (RAGE). Green lines indicate the nature of the association is correlation, and black lines indicate protein-protein interactions. Genes in green boxes are positively correlated with *AZU1*. Genes in blue boxes encode for proteins that interact with at least two other proteins in the network. Three of the genes shown in green boxes are S100 calgranulin genes (S100A8, S100A9, and S100A12), that encode for calcium binding proteins and are RAGE ligands. *S100A8*, *S100A9*, and *S100A12* correlated with *AZU1* with Pearson’s correlation coefficient values (measures of linear correlations) of 0.41, 0.37, and 0.38, respectively. The expression of *ELANE* (neutrophil elastase), *CTSG* (cathepsin G), and *CAMP* (cathelicidin antimicrobial peptide) positively correlated with *AZU1* (Pearson’s correlation coefficient values of 0.72, 0.62, and 0.43, respectively). Genes that positively correlated with *AZU1* had protein-protein interactions with genes involved in blood clotting (*F5*, *F2RL1*, *F2R*, *KNG1*), protease inhibition (*SERPIN*s), cell adhesion (*THBS1*, *SDC*, *VCAM1*), chemotaxis (*CXCR4*, *CXCL12*), Aβ transport (*LRP1*), and cytoskeletal structure (*CAPZA1*, *DES*, *GFAP*). A protein-protein interaction between *S100A12* and *AGER* is shown. Genes encoding for other ligands of RAGE (*S100B*, *S100P*, *S100A4*, *S100A1*) were also associated with *AZU1* based on the interactions in the network.

To determine the potential function of CAP37 in the brains of patients with AD, we first conducted an analysis to investigate which genes were within the “genetic neighborhood” of CAP37. GAMMA [11] was used to analyze transcriptomic data and identify genes highly correlated with CAP37. Then, protein-protein interactions between highly correlated genes were identified using the Human Proteome Reference Database. The subset of this network surrounding CAP37 (encoded by the gene *AZU1*) and *AGER* is shown in Fig 1. Green lines indicate the nature of the association is correlation, and black lines indicate protein-protein interactions. Genes that positively correlated with *AZU1* are represented by green boxes, and blue boxes represent protein-protein interactions between respective encoded genes. The genes encoding neutrophil proteins, neutrophil elastase (*ELANE*), cathepsin G (*CTSG*), and cathelicidin antimicrobial peptide (*CAMP*), positively correlated with *AZU1*. In addition, three of the S100-subtype calgranulin genes (*S100A12*, *S100A8*, and *S100A9*) correlated with *AZU1* (Fig 1). The S100 proteins are calcium binding proteins involved in various cell processes [27]. Similar to CAP37, these particular S100 proteins are expressed constitutively in neutrophils, mediate inflammatory responses, and have antimicrobial activities [27–29]. *AGER*, which encodes RAGE, was revealed by the Human Proteome Reference Database as one of the proteins interacting with S100A12. The literature indicates that S100A8, S100A9, and S100A12 are RAGE ligands that activate various cell responses by signaling through RAGE [27, 30]. Other proteins interacting with the S100 proteins included proteins involved in tumor suppression (*TP53*), transcriptional regulation (*LRIF1*), ubiquitination (*CACYBP*), and neurotransmitter release (*UNC119*). Due to the high correlation and functional similarities of CAP37 with the S100 proteins, we investigated whether CAP37 and two other neutrophil proteins, neutrophil elastase and cathepsin G, also interacted with RAGE.

### RAGE binds specifically to CAP37, neutrophil elastase, and cathepsin G with different affinities

**Fig 2:**
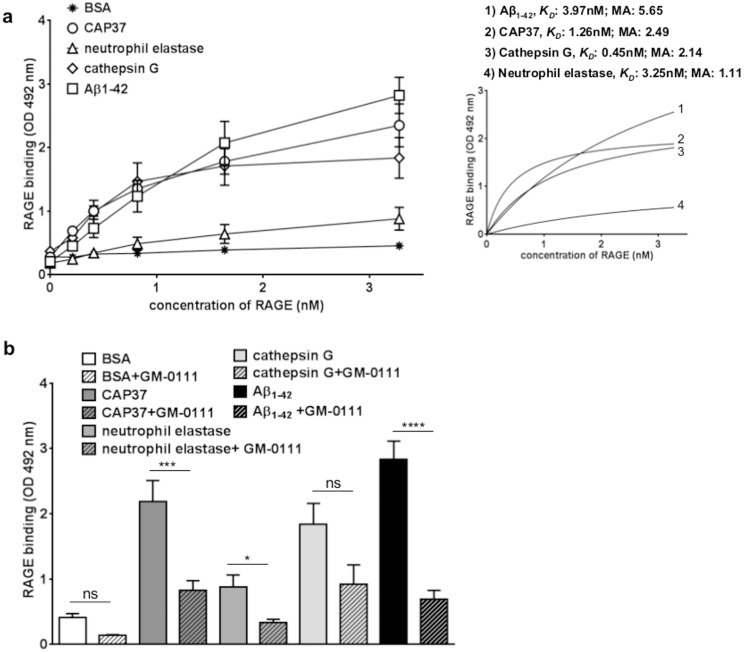
RAGE binds specifically to CAP37, neutrophil elastase, and cathepsin G, but with different affinities. (a) Graph shows the binding of RAGE to BSA (stars), CAP37 (circles), neutrophil elastase (triangles), cathepsin G (diamonds), and Aβ_1-42_(squares). BSA: n = 20, CAP37: n = 11, neutrophil elastase: n = 7, cathepsin G: n = 9, Aβ_1–42_: n = 12. Data are mean ± SEM of results. *K*_*D*_ values and maximum absorbance (MA) values were obtained after subtracting BSA as background and fitting values to curves of nonlinear regression (see insert). (b) Graph shows binding of RAGE (3.3 nM) to BSA (white bars), CAP37 (dark gray bars), neutrophil elastase (medium gray bars), cathepsin G (light gray bars), and Aβ_1-42_(black bars) after pre-incubating in the absence (solid bars) or presence (striped bars) of GM-0111. BSA: n = 20, BSA+GM-0111: n = 3, CAP37: n = 11, CAP37+GM-0111: n = 10, neutrophil elastase: n = 7, neutrophil elastase+GM-0111: n = 6, cathepsin G: n = 9, cathepsin G+GM-0111: n = 4, Aβ_1–42_: n = 12, Aβ_1–42_ +GM-0111: n = 9. Student’s unpaired *t* tests were performed to compare binding of RAGE to each protein in the presence and absence of GM-0111. Data are mean ± SEM of results. *p<0.05,***p<0.001, ****p<0.0001 when comparing binding with and without GM-0111.

ELISAs were used to determine binding of RAGE to the neutrophil proteins CAP37, neutrophil elastase, and cathepsin G. RAGE was added at increasing concentrations to wells coated with the respective neutrophil proteins. The binding of RAGE to Aβ_1–42_ was measured as a positive control since Aβ_1–42_ is an established ligand of RAGE [14]. Binding of RAGE to BSA was measured as a control for non-specific protein binding. Dissociation constant (*K*_*D*_) values, which indicate the protein concentration required to reach half-maximal binding at equilibrium and reflect the binding affinity, were computed for binding of RAGE to each protein. The maximum absorbance values, which reflect the maximum number of binding sites, were also determined. Results demonstrated significantly more binding (p<0.0001) of RAGE to CAP37 than to BSA at all concentrations of RAGE tested (Fig 2a, Table 1). RAGE bound to CAP37 with a *K*_*D*_ value of 1.26 nM and a maximum absorbance value of 2.49 (Fig 2a, insert). Binding of RAGE to neutrophil elastase was also significantly higher (p<0.01) than binding to BSA, but only at the two highest concentrations of RAGE tested (Fig 2a, Table 1). A *K*_*D*_ value of 3.25 nM and a maximum absorbance value of 1.11 was computed for RAGE binding to neutrophil elastase (Fig 2a, insert). RAGE binding to cathepsin G was significantly higher (p<0.0001) than binding to BSA at all concentrations of RAGE tested (Fig 2a, Table 1), and a *K*_*D*_ value of 0.45 nM and a maximum absorbance value of 2.14 were obtained (Fig 2a, insert). Among the three neutrophil proteins, CAP37 bound to the highest number of RAGE molecules (based on maximum absorbance values). RAGE binding to cathepsin G exhibited the lowest *K*_*D*_ value and hence the highest affinity. As expected, results showed significantly higher binding (p<0.0001) of RAGE to Aβ_1–42_ compared to BSA at all concentrations of RAGE tested (Fig 2a, Table 1). A *K*_*D*_ value of 3.97 nM and maximum absorbance value of 5.65 was calculated for RAGE binding to Aβ_1–42_ (Fig 2a, insert).

**Table 1:** RAGE binding to Aβ_1–42_, CAP37, neutrophil elastase, and cathepsin G.

RAGE (nM)	Log binding			
	Aβ_1–42_	CAP37	Neutrophil elastase	Cathepsin G
0	-0.726±0.054 ns p = 0.851	-0.623±0.077 ns p = 0.979	-0.756±0.060 ns p = 0.744	-0.426±0.058 ns p = 0.105
0.21	-0.387±0.058* p = 0.011	-0.174±0.031**** p<0.0001	-0.629±0.055 ns p = 0.10	-0.264±0.062*** p = 0.0002
0.42	-0.214±0.076**** p<0.0001	-0.012±0.034**** p<0.0001	-0.510±0.087 ns p = 0.859	-0.078±0.0089**** p<0.0001
0.83	-0.013±0.094**** p<0.0001	0.112±0.041**** p<0.0001	-0.376±0.101 ns p = 0.136	0.101±0.084**** p<0.0001
1.67	0.238±0.088**** p<0.0001	0.228±0.043**** p<0.0001	-0.274±0.113* p = 0.046	0.166±0.092**** p<0.0001
3.33	0.425±0.048**** p<0.0001	0.325±0.064**** p<0.0001	-0.121±0.104** p = 0.003	0.192±0.096**** p<0.0001

Log RAGE binding is shown as mean± SEM. A two-way ANOVA was performed with Dunnett’s multiple comparisons test so that binding of RAGE to each protein was compared to binding of RAGE to BSA.

*p<0.05

**p<0.01

***p<0.001

****p<0.0001

To determine whether or not binding of RAGE to the neutrophil proteins could be inhibited by a specific inhibitor of RAGE, we measured binding of RAGE to the neutrophil proteins in the presence of the RAGE antagonist, GM-0111. When GM-0111 was pre-incubated with RAGE, binding of RAGE to CAP37 was significantly reduced (Fig 2b, Table 2B). Pre-incubation with GM-0111 also significantly reduced the binding of RAGE to neutrophil elastase, but binding was only reduced when the highest concentration of RAGE was added (Fig 2b, Table 2B). Although binding of RAGE to cathepsin G was observed with high affinity, pre-incubation of RAGE with GM-0111 did not significantly reduce binding of RAGE to cathepsin G (Fig 2b, Table 2B). As would be expected, GM-0111 significantly reduced the binding of RAGE to Aβ_1–42_ but not to BSA (Fig 2b, Table 2A).

**Table 2:** Inhibition of RAGE binding BSA, Aβ_1–42_, CAP37, neutrophil elastase, and cathepsin G with GM0-111.

**A**								
**RAGE (nM)**	**BSA**		**Aβ**_**1–42**_					
	RAGE binding (OD 492 nm)		RAGE binding (OD 492 nm)					
	-GM-0111	+GM-0111	-GM-0111		+GM-0111			
0	0.260±0.044	0.117±0.006 ns p = 0.229	0.208±0.034		0.190±0.021 ns p = 0.690			
0.21	0.255±0.030	0.125±0.013 ns p = 0.109	0.451±0.057		0.284±0.040 * p = 0.038			
0.42	0.296±0.036	0.128±0.013 ns p = 0.092	0.730±0.144		0.339±0.052 * p = 0.036			
0.83	0.307±0.044	0.127±0.015 ns p = 0.137	1.234±0.247		0.448±0.072 * p = 0.015			
1.67	0.350±0.046	0.151±0.018 ns p = 0.118	2.086±0.335		0.540±0.091 *** p = 0.0009			
3.33	0.412±0.057	0.138±0.008 ns p = 0.082	2.834±0.275		0.693±0.133 **** p<0.0001			
**B**								
**RAGE (nM)**	**CAP37**			**Neutrophil elastase**		**Cathepsin G**		
	RAGE binding (OD 492 nm)			RAGE binding (OD 492 nm)		RAGE binding (OD 492 nm)		
	-GM-0111	+GM-0111		-GM-0111	+GM-0111	-GM-0111		+GM-0111
0	0.277±0.047	0.340±0.079 ns p = 0.499		0.183± 0.019	0.183±0.021 ns p = 0.972	0.369±0.043		0.489±0.101 ns p = 0.214
0.21	0.688±0.052	0.355±0.042 **** p<0.0001		0.246± 0.027	0.187±0.024 ns p = 0.137	0.583±0.065		0.461±0.067 ns p = 0.283
0.42	1.002±0.082	0.409±0.051 **** p<0.0001		0.343±0.055	0.208±0.029 ns p = 0.063	0.979±0.192		0.586±0.104 ns p = 0.220
0.83	1.353±0.134	0.563±0.076 **** p<0.0001		0.488±0.100	0.254±0.036 ns p = 0.065	1.467±0.293		0.839±0.181 ns p = 0.204
1.67	1.783±0.201	0.648±0.111 *** p = 0.0001		0.641±0.148	0.294±0.041 ns p = 0.059	1.709±0.302		0.942±0.241 ns p = 0.145
3.33	2.349±0.338	0.826±0.146 *** p = 0.0007		0.882±0.179	0.332±0.050 * p = 0.019	1.838±0.320		0.924±0.292 ns p = 0.109

RAGE binding is shown as mean± SEM. Student’s unpaired *t* tests were performed to compare RAGE binding in the presence or absence of RAGE antagonist, GM-0111.

*p<0.05

**p<0.01

***p<0.001

****p<0.0001

**Fig 3:**
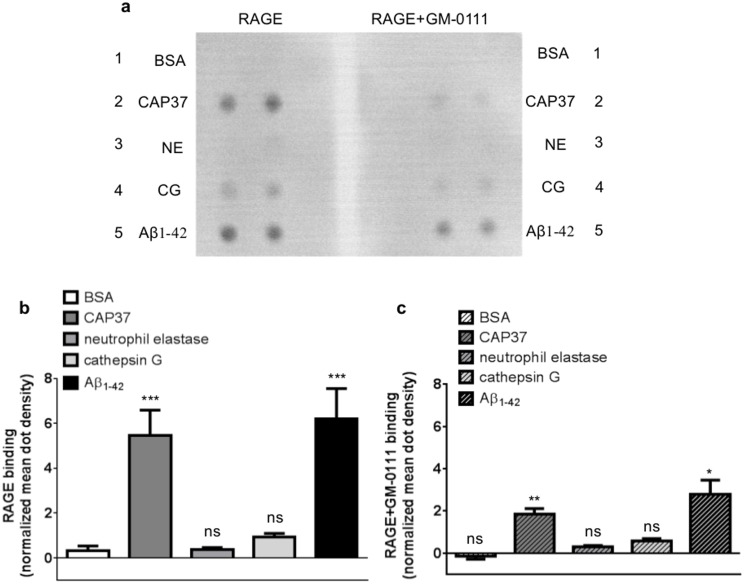
Far-Western dot blotting confirms the binding of RAGE to neutrophil proteins and inhibition of binding with GM-0111. (a) Far-Western dot blots show the binding of RAGE to BSA (row 1), CAP37 (row 2), neutrophil elastase (NE, row 3), cathepsin G (CG, row 4), and Aβ_1–42_ (row 5) when RAGE was pre-incubated in the absence (left) or presence (right) of the RAGE inhibitor, GM-0111. Proteins were spotted in duplicate on each blot. (b) Histogram quantification of RAGE binding to BSA, CAP37, neutrophil elastase, cathepsin G, and Aβ_1–42_ is represented by the white bar, dark gray bar, medium gray bar, light gray bar, and black bar, respectively. BSA: n = 9, CAP37: n = 9, neutrophil elastase: n = 6, cathepsin G: n = 6, Aβ_1–42_: n = 8. Data are mean ± SEM of results. Mean dot densities were normalized to the mean dot densities of ponceau S staining for each dot. A Kruskal-Wallis test was performed with Dunn’s multiple comparisons test to compare the binding of RAGE to each neutrophil protein with the binding of RAGE to BSA. (c) Histogram quantification of RAGE (pre-incubated with GM-0111) binding to BSA, CAP37, neutrophil elastase, cathepsin G, and Aβ_1–42_ is represented by the striped white bar, striped dark gray bar, striped medium gray bar, striped light gray bar, and striped black bar, respectively. BSA+GM-0111: n = 8, CAP37+GM-0111: n = 8, neutrophil elastase+GM-0111: n = 5, cathepsin G+GM-0111: n = 5, Aβ_1–42_+GM-0111: n = 7. Data are mean ± SEM of results. Mean dot densities were normalized to the mean dot densities of ponceau S staining for each dot. Asterisks (*) indicate significant results from student’s unpaired *t* tests, which were used to compare binding of RAGE to each protein in the absence and presence of GM-0111.

Far Western dot blot analysis was used to confirm the binding of RAGE to neutrophil proteins. Similar to results obtained with ELISAs, far-Western dot blot analysis demonstrated strong binding of RAGE to Aβ_1–42_ and CAP37 (Fig 3a). Binding of RAGE to cathepsin G was also observed. However, binding of RAGE to cathepsin G was not significantly higher than RAGE binding to BSA. We did not detect visible binding of RAGE to neutrophil elastase or BSA using this approach. These results are congruent with ELISA results, which also showed the lowest binding of RAGE to neutrophil elastase and BSA. Mean dot density quantification using Image J software revealed that binding of RAGE to Aβ_1–42_ and CAP37 was significantly higher than binding of RAGE to BSA (Fig 3b). Similar to ELISAs, GM-0111 also significantly decreased the binding of RAGE to Aβ_1–42_ and CAP37 in far-Western dot blot experiments (Fig 3c).

### CAP37 and neutrophil elastase decrease the amyloid beta-RAGE interaction by distinct mechanisms

**Fig 4:**
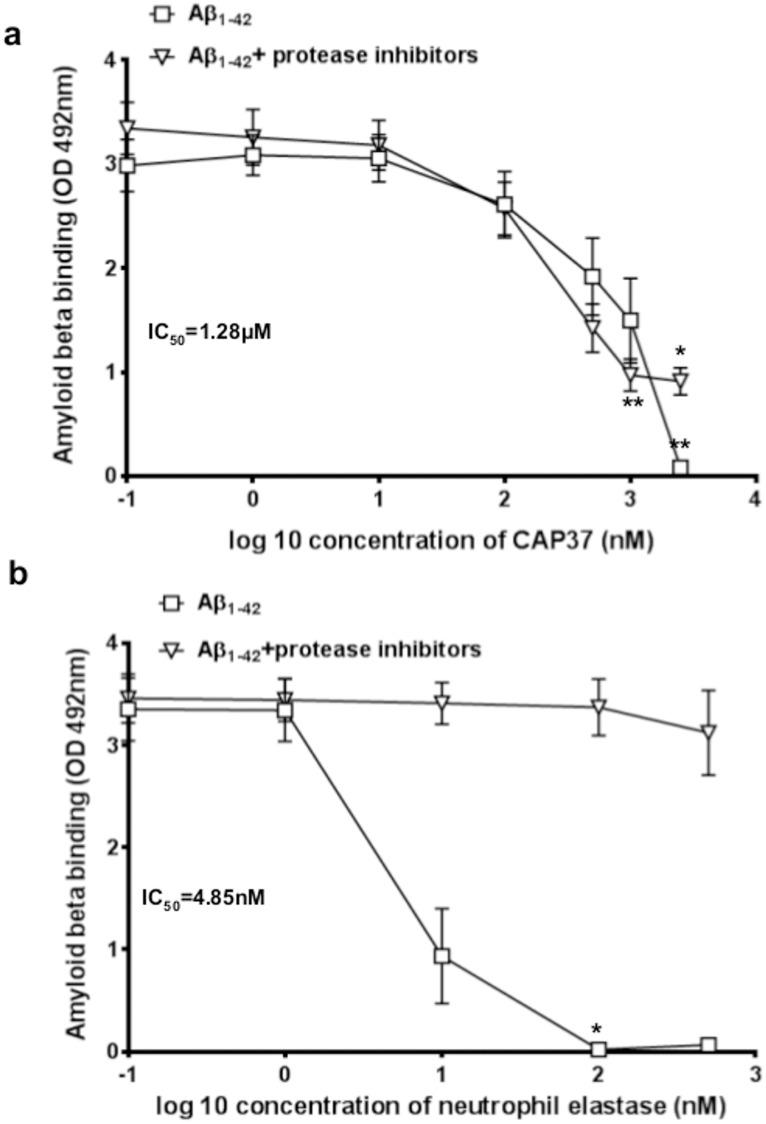
Neutrophil proteins inhibit Aβ_1–42_ binding to RAGE. (a) Graph shows binding of Aβ_1–42_ to RAGE when CAP37 was added simultaneously to wells at increasing concentrations in the presence (inverted triangles) or absence (squares) of protease inhibitors. An IC_50_ value of 1.28 μM was computed for inhibition of Aβ_1–42_ binding to RAGE by CAP37. Two separate Kruskal-Wallis tests were performed for Aβ_1–42_ binding in the presence and absence of protease inhibitors. A Dunn’s multiple comparisons test was performed for each separate Kruskal-Wallis test to compare binding of Aβ_1–42_ to RAGE in the presence of increasing concentrations of CAP37. Data are mean ± SEM of results. *p<0.05, **p<0.01. (b) Graph shows binding of Aβ_1–42_ to RAGE when neutrophil elastase was added simultaneously to wells at increasing concentrations in the presence (inverted triangles) or absence (squares) of protease inhibitors. An IC_50_ value of 4.85 nM was computed for inhibition of Aβ_1–42_ binding to RAGE by neutrophil elastase in the absence of protease inhibitors. Two separate Kruskal-Wallis tests were performed for Aβ_1–42_ binding in the presence and absence of protease inhibitors. A Dunn’s multiple comparisons test was performed for each separate Kruskal-Wallis test to compare binding of Aβ_1–42_ to RAGE in the presence of increasing concentrations of neutrophil elastase. No inhibition occurred in the presence of protease inhibitors. Data are mean ± SEM of results. *p<0.05.

Upon observing that the binding of CAP37, neutrophil elastase, and Aβ_1–42_ to RAGE could be inhibited by the antagonist, GM-0111, we next sought to determine if either CAP37 or neutrophil elastase could compete with Aβ_1–42_ for the same binding site on RAGE. Results obtained from ELISAs, demonstrated that when Aβ_1–42_ and CAP37 were added simultaneously to wells containing RAGE, CAP37 significantly decreased the binding of Aβ_1–42_ to RAGE in a dose-dependent manner (Fig 4a). CAP37 inhibited the binding of Aβ_1–42_ to RAGE with a half maximal inhibitory concentration (IC_50_) value of 1.28 μM. Under these conditions, the concentration of CAP37 (1.28 μM) is ≈ 100-fold higher than the concentration of Aβ_1–42_ (11 nM). Neutrophil elastase significantly decreased the binding of Aβ_1–42_ to RAGE with a very low IC_50_ value of 4.58 nM (Fig 4b). Since we did not observe strong binding of RAGE to neutrophil elastase (Fig 2a), inhibition by neutrophil elastase with such a low IC_50_ value led us to question if this inhibition was indeed due to displacement of Aβ_1–42_ binding to RAGE or if it may have been due to the proteolytic activity of neutrophil elastase. To determine this, we measured binding and inhibition of the Aβ_1-42_- RAGE interaction in the presence of a protease inhibitor cocktail. Interestingly, addition of protease inhibitors completely prevented neutrophil elastase from inhibiting Aβ_1–42_ binding to RAGE (Fig 4b). This indicated that neutrophil elastase was not likely competitively displacing Aβ_1–42_ bound to RAGE, but was more likely degrading the Aβ_1–42_ to prevent it from binding to RAGE. The protease inhibitors did not prevent CAP37 from inhibiting the Aβ_1-42-_RAGE interaction (Fig 4a). However, maximal inhibition in the absence of protease inhibitors was complete (no Aβ binding to RAGE at the highest dose of CAP37), and only partial in the presence of protease inhibitors. This raised the possibility that CAP37 also had some enzymatic activity against Aβ_1–42_, although most likely less potent compared to neutrophil elastase.

**Fig 5:**
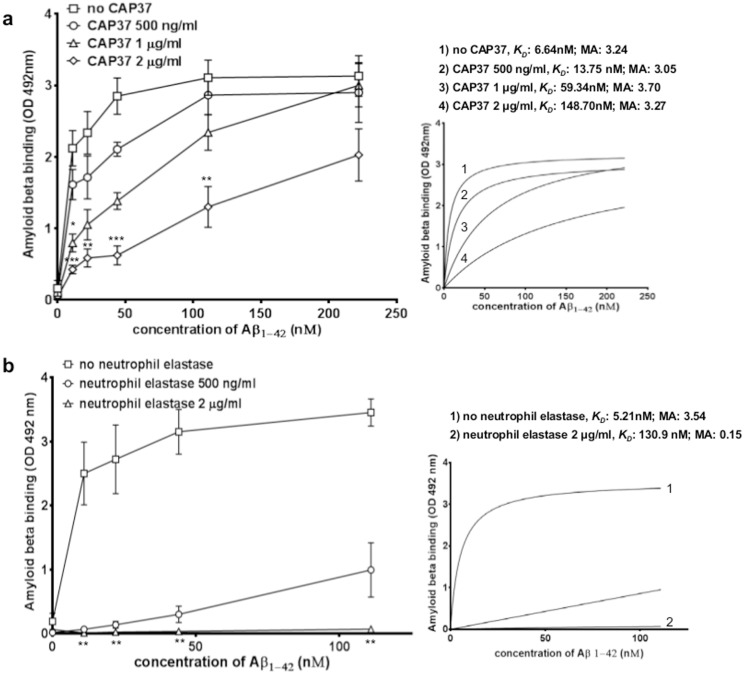
Neutrophil proteins pre-incubated with Aβ_1–42_ inhibit the binding of Aβ_1–42_ to RAGE. (a) Graph shows binding of Aβ_1–42_ to RAGE when Aβ_1–42_ was pre-incubated alone (squares) and a dose-dependent reduction of Aβ_1–42_ binding to RAGE when Aβ_1–42_ pre-incubated with 0.5 μg/ml (21 nM, circles), 1 μg/ml (42 nM, triangles), or 2 μg/ml CAP37 (83 nM, diamonds). No CAP37: n = 5, +CAP37 0.5 μg/ml: n = 3, +CAP37 1 μg/ml: n = 4, +CAP37 2 μg/ml: n = 4. BSA was subtracted as background from all curves. Separate Kruskal-Wallis tests were performed to analyze binding at each concentration of Aβ_1–42_. A Dunn’s multiple comparisons test was performed for each Kruskal-Wallis test to compare binding of Aβ_1–42_ to RAGE when Aβ_1–42_ pre-incubated with increasing concentrations of CAP37. *p<0.05,**p<0.01, ***p<0.001. *K*_*D*_ values and maximum absorbance (MA) values were computed after fitting values to curves of nonlinear regression (see insert). (b) Graph shows binding of Aβ_1–42_ to RAGE when Aβ_1–42_ pre-incubated alone (squares) and a dose-dependent reduction of the Aβ_1–42_ binding RAGE when Aβ_1–42_ pre-incubated with 0.5 μg/ml (20 nM, circles) or 2 μg/ml neutrophil elastase (80 nM, triangles). No neutrophil elastase: n = 5, +neutrophil elastase 0.5 μg/ml: n = 4, +neutrophil elastase 2 μg/ml: n = 5. BSA was subtracted as background from all curves. Separate Kruskal-Wallis tests were performed to analyze binding at each concentration of Aβ_1–42_. A Dunn’s multiple comparisons test was performed for each Kruskal-Wallis test to compare binding of Aβ_1–42_ to RAGE when Aβ_1–42_ pre-incubated with increasing concentrations of neutrophil elastase.**p<0.01. *K*_*D*_ values and maximum absorbance (MA) were computed after fitting values to curves of nonlinear regression (see insert).

Based on these findings, we next questioned whether the Aβ_1-42_-RAGE interaction could be disrupted through direct interactions of the neutrophil proteins with Aβ_1–42_. To determine this, we performed ELISAs in which CAP37 and neutrophil elastase were pre-incubated with Aβ_1–42_ before adding Aβ_1–42_ to wells containing RAGE. Interestingly, when pre-incubated with Aβ_1–42_, both CAP37 (Fig 5a) and neutrophil elastase (Fig 5b) significantly reduced the interaction of Aβ_1–42_ with RAGE in a dose dependent manner. CAP37 (at 2 μg/ml) increased the *K*_*D*_ value for Aβ_1–42_ binding to RAGE from 6.64 nM to 148.70 nM (Fig 5a, insert) but did not change the maximum absorbance value (3.24 to 3.27 OD 492 nm), whereas, neutrophil elastase (at 2 μg/ml) increased the *K*_*D*_ value from 5.21 nM to 130.90 nM (Fig 5b, insert) and reduced the maximum absorbance value (3.54 to 0.15 OD 492 nm). This indicates that CAP37 and neutrophil elastase likely inhibit the interaction of Aβ_1–42_ with RAGE through distinct mechanisms, and the results suggest two mechanisms of inhibition were possible. CAP37 and neutrophil elastase could be proteolytically degrading the Aβ_1–42_, preventing it from binding to RAGE. Alternatively, CAP37 and neutrophil elastase could be binding Aβ_1–42_ with a high affinity, and therefore, sequestering it away from RAGE.

### Aβ_1–42_ binds to CAP37, neutrophil elastase, and cathepsin G with different affinities

**Fig 6:**
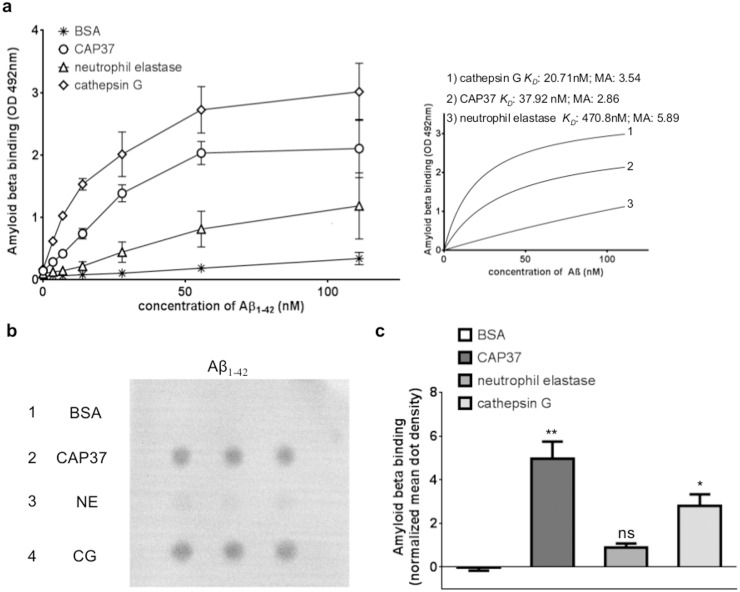
Amyloid beta binds to CAP37, neutrophil elastase, and cathepsin G with different affinities. (a) Graph shows binding of Aβ_1–42_ to CAP37 (circles), neutrophil elastase (triangles), cathepsin G (diamonds), and BSA (stars). n = 3 for all proteins. Data are mean ± SEM of results. *K*_*D*_ values and maximum absorbance (MA) values were obtained after subtracting BSA as background and fitting values to curves of nonlinear regression (see insert). (b) Far-Western dot blot shows the binding of Aβ_1–42_ to BSA (row 1), CAP37 (row 2), neutrophil elastase (NE, row 3), and cathepsin G (CG, row 4). Proteins were spotted in triplicate across each row. (c) Histogram quantification of Aβ_1–42_ binding BSA, CAP37, neutrophil elastase, and cathepsin G, represented by the white bar, dark gray bar, medium gray bar, and light gray bar, respectively. n = 4 for all proteins. Data are mean ± SEM of results. Mean dot densities were normalized to the mean dot densities of ponceau S staining for each dot. A Kruskal-Wallis test was performed with Dunn’s multiple comparisons test to compare the binding of Aβ_1–42_ to each neutrophil protein with the binding of Aβ_1–42_ to BSA. *p<0.05, **p<0.01.

To explore the possibility that neutrophil proteins could bind and sequester Aβ_1–42_, we tested for specific binding of Aβ_1–42_ to CAP37, neutrophil elastase, or cathepsin G. This was achieved by performing an ELISA in which Aβ_1–42_ was added at increasing concentrations to wells containing 500 ng of CAP37, neutrophil elastase, or cathepsin G. This experiment was performed in the presence of protease inhibitors to establish binding in absence of proteolytic activity. Binding of Aβ_1–42_ to BSA was measured as a control for non-specific protein binding. We observed binding of Aβ_1–42_ to all three neutrophil proteins; we observed significantly higher (p<0.01 to p<0.0001) binding of Aβ_1–42_ to CAP37 compared to BSA at all doses of Aβ_1–42_ tested (Fig 6, Table 3). Aβ_1–42_ bound to CAP37 with a calculated *K*_*D*_ value of 37.92 nM and a maximum absorbance value of 2.86 (Fig 6, insert). Binding of Aβ_1–42_ to cathepsin G was also significantly higher than binding to BSA at all concentrations of Aβ_1–42_ (Fig 6, Table 3). An absorbance background, likely due to a non-specific reaction between the antibody for Aβ_1–42_ and CAP37 and cathepsin G was observed in the absence of Aβ_1–42_ (Table 3). A *K*_*D*_ value of 20.71 nM and maximum absorbance value of 3.54 was computed for Aβ_1–42_ binding to cathepsin G (Fig 6, insert). Binding of Aβ_1–42_ to neutrophil elastase (Fig 6, Table 3) was lower, but was significant starting at an Aβ_1–42_ concentration of 6.93 nM. A higher *K*_*D*_ value of 470.80 nM and a maximum absorbance value of 5.89 was computed for Aβ_1–42_ binding to neutrophil elastase (Fig 6, insert).

**Table 3:** Aβ_1–42_ binding to CAP37, neutrophil elastase, and cathepsin G.

Aβ_1–42_ (nM)	Log binding		
	CAP37	Neutrophil elastase	Cathepsin G
0	-0.907±0.139** p = 0.005	-1.061±0.114 ns p = 0.119	-0.884±0.103 *** p = 0.0004
3.47	-0.556±0.047 **** p<0.0001	-0.961±0.139 ns p = 0.169	-0.368±0.157 **** p<0.0001
6.93	-0.389±0.051 **** p<0.0001	-0.890±0.127 * p = 0.0064	-0.152±0.162 **** p<0.0001
13.89	-0.138±0.047 **** p<0.0001	-0.720±0.151 ** p = 0.0064	0.019±0.165 **** p<0.0001
27.78	0.137±0.043 **** p<0.0001	-0.414±0.154 **** p<0.0001	0.151±0.149 **** p<0.0001
55.56	0.304±0.039 **** p<0.0001	-0.146±0.153 **** p<0.0001	0.310±0.125 **** p<0.0001
111	0.377±0.075 **** p<0.0001	0.123±0.176 *** p = 0.0004	0.541±0.002 **** p<0.0001

Log Aβ_1–42_ binding is shown as mean± SEM. A two-way ANOVA was performed with Dunnett’s multiple comparisons test so that binding of Aβ_1–42_ to each protein was compared to binding of Aβ_1–42_ to BSA.

*p<0.05

**p<0.01

***p<0.001

****p<0.0001

Far-Western dot blot analysis was used to confirm the binding of Aβ_1–42_ to neutrophil proteins. Similar to results obtained with ELISAs, far-Western dot blot analysis demonstrated strong binding of Aβ_1–42_ to CAP37 and cathepsin G and low binding to neutrophil elastase (Fig 6b). No binding of Aβ_1–42_ to BSA was visible. Mean dot density quantification using Image J software revealed that binding of Aβ_1–42_ to CAP37 and cathepsin G was significantly higher than binding of Aβ_1–42_ to BSA (Fig 6c). However, binding of Aβ_1–42_ to neutrophil elastase was not significantly higher than binding of Aβ_1–42_ to BSA.

### CAP37, neutrophil elastase, and cathepsin G cleave Aβ_1–42_

**Fig 7:**
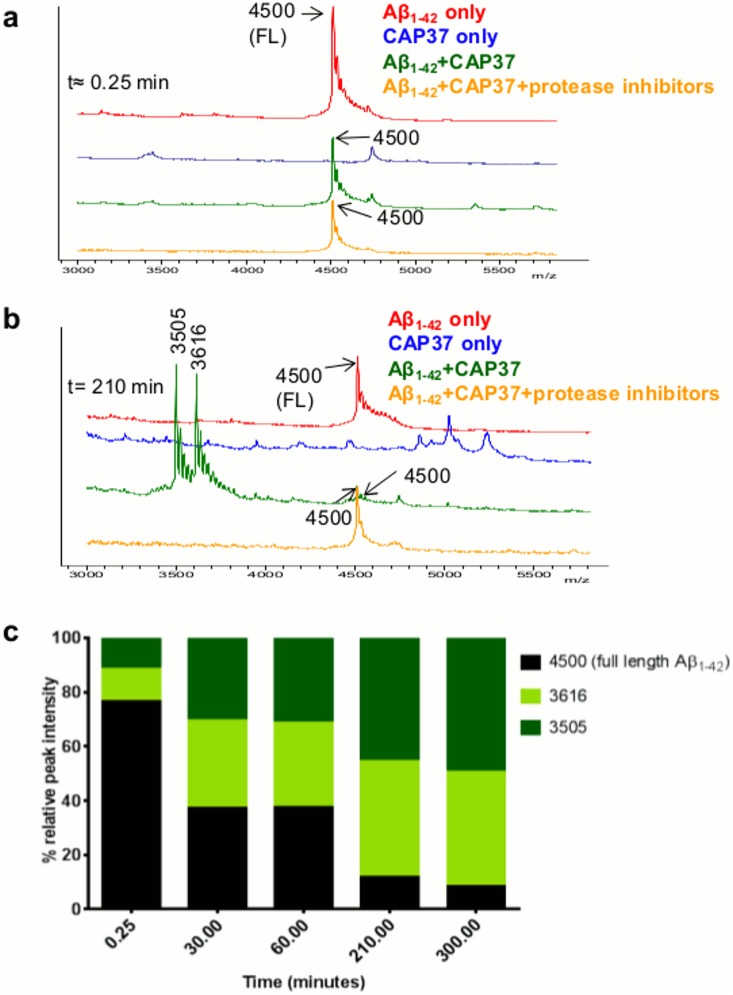
CAP37 slowly cleaves Aβ_1–42_. (a) MALDI-TOF spectra of Aβ_1–42_ that incubated alone (red), with CAP37 (green), or with CAP37+protease inhibitors (orange) at t≈0.25 min. Peaks are labeled with mass-to-charge ratios (m/z), and mass is in Daltons (Da). A peak of 4500 Da, which represents full length (FL) Aβ_1–42_, appears in all Aβ_1–42_ spectra. CAP37 incubated alone is shown in blue, and no peak at 4500 Da is exhibited by CAP37. (b) MALDI-TOF spectra at t = 210 min. Note the reduced size of the full Aβ_1–42_ peak and the presence of two large Aβ_1–42_ fragment peaks at 3616 Da and 3505 Da when Aβ_1–42_ incubated with CAP37. (c) Graph shows % relative peak intensity of Aβ_1–42_ and its fragments (intact Aβ_1–42_ + Aβ_1–42_ fragments) analyzed at t≈ 0.25 min, t = 30 min, t = 60 min, t = 210 min, and t = 300 min. Data are representative of 2 independent experiments.

**Fig 8:**
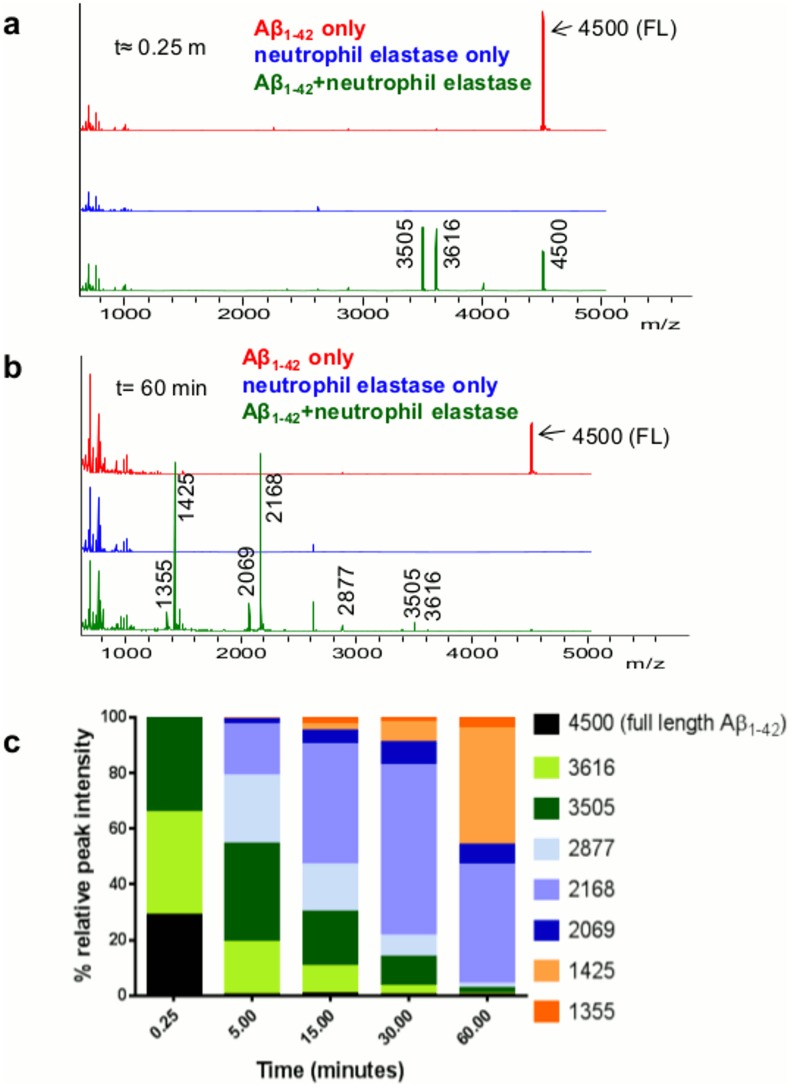
Neutrophil elastase rapidly cleaves Aβ_1–42_. (a) MALDI-TOF spectra of Aβ_1–42_ incubated alone (red) or with neutrophil elastase (green) at t≈0.25 min. Peaks are labeled with mass-to-charge ratios (m/z), and mass is in Daltons (Da). A peak of 4500 Da, which represents intact Aβ_1–42_, appears in all Aβ_1–42_ spectra. Two fragment peaks at 3616 Da and 3505 Da are already present after incubation with neutrophil elastase at t≈0.25 min. Neutrophil elastase incubated alone is shown in blue, and no peak at 4500 Da is exhibited by neutrophil elastase. (b) MALDI-TOF spectra at t = 60 min. Note the reduced size of the full Aβ_1–42_ peak and the peaks at 3616 Da and 3605 Da and the presence of 5 additional fragment peaks at 2877 Da, 2168 Da, 2069 Da, 1425 Da, and 1355 Da when Aβ_1–42_ incubated with neutrophil elastase (green spectra). (c) Graph shows % relative peak intensity of Aβ_1–42_ and its fragments (intact Aβ_1–42_ + Aβ_1–42_ fragments) analyzed at t≈ 0.25 min, t = 5 min, t = 15 min, t = 30 min, and t = 60 min. Data are representative of 3 independent experiments.

**Fig 9:**
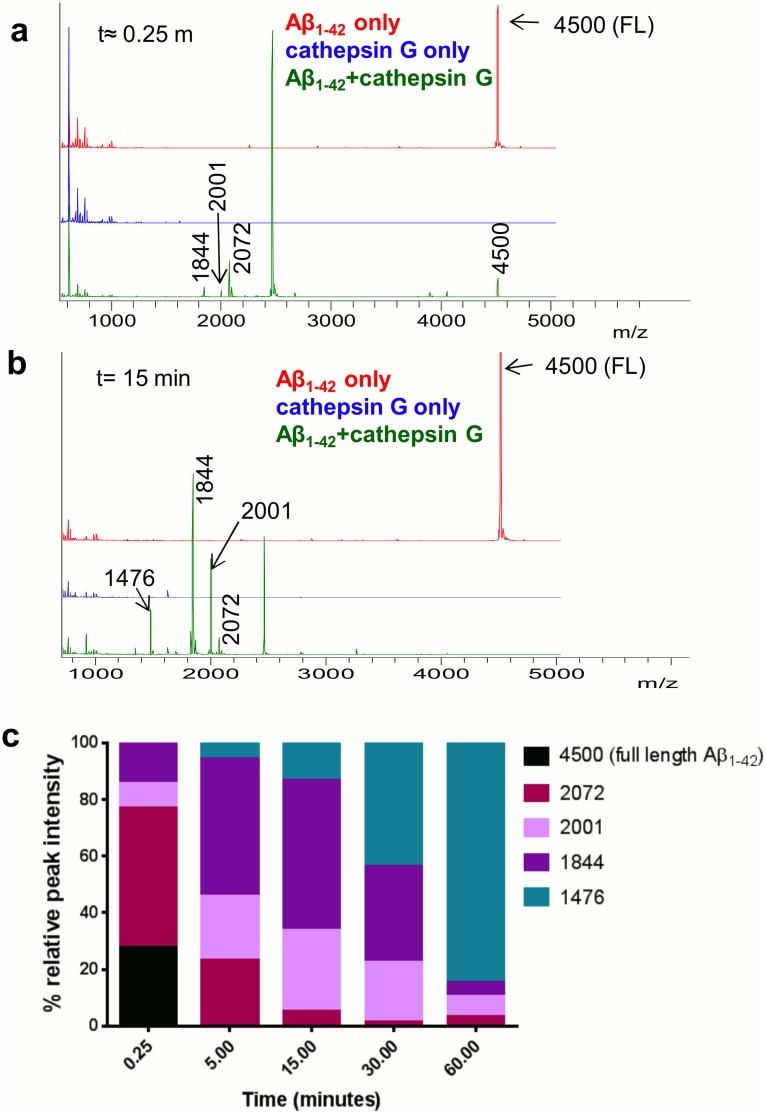
Cathepsin G rapidly cleaves Aβ_1–42_. (a) MALDI-TOF spectra of Aβ_1–42_ incubated alone (red) or with cathepsin G (green) at t≈0.25 min. Peaks are labeled with mass-to-charge ratios (m/z), and mass is in Daltons (Da). A peak of 4500 Da, which represents intact Aβ_1–42_, appears in all Aβ_1–42_ spectra. Peaks predicted to represent Aβ_1–42_ fragments at 2072 Da, 2001 Da, and 1844 Da are already present after incubation with cathepsin G at t≈0.25 min (green). Cathepsin G incubated alone is shown in blue, and no peak at 4500 Da is exhibited by cathepsin G. (b) MALDI-TOF spectra at t = 15 min. Note the absence of the full Aβ_1–42_ peak and the presence of products at 2072 Da, 2001 Da, and 1844 Da as well as an additional fragment peak at 1476 Da when Aβ_1–42_ incubated with cathepsin G (green). (c) Graph shows relative % peak intensity of Aβ_1–42_ and its fragments (intact Aβ_1–42_ + Aβ_1–42_ fragments at each time point) analyzed at t≈ 0.25 min, t = 5 min, t = 15 min, t = 30 min, and t = 60 min. Data are representative of 3 independent experiments.

Our results suggest that neutrophil proteins could inhibit the binding of Aβ_1–42_ to RAGE through direct binding and sequestering of Aβ_1–42_. We also observed that enzymatic degradation of Aβ_1–42_ could be inhibited by protease inhibitors, but without the inhibitors, the Aβ_1–42_ was degraded and binding to RAGE eliminated. In this experiment, we aimed to establish unequivocally the cleavage of Aβ_1–42_. We performed MALDI-TOF MS on Aβ that was incubated with CAP37 (Fig 7) in the presence and absence of protease inhibitors at room temperature up to 5 h (t≈0.25 min to t = 300 min), or with neutrophil elastase (Fig 8) or cathepsin G (Fig 9) at room temperature for up to 1 h (t≈0.25 m to t = 60 min). Proteins were incubated at room temperature rather than 37°C due to the formation of Aβ_1–42_ aggregates at 37°C. Immediately after adding CAP37 (t≈0.25 min), we observed a peak at 4500 Da corresponding to the full length Aβ_1–42_ (Fig 7a), which represented 76.65% of the total intensity (Fig 7c) of Aβ peaks (intact full peptide + fragments generated). As shown in Fig 7b, after Aβ_1–42_ incubated for 210 min with CAP37, the 4500 Da peak was smaller than when amyloid beta incubated alone or incubated with CAP37 in the presence of protease inhibitors. In addition, two large peaks appeared at 3616 Da and 3505 Da after incubation with CAP37, which were not present when Aβ_1–42_ was incubated alone or with CAP37 and protease inhibitors. No peaks corresponding to the molecular weight of Aβ_1–42_ or the products of Aβ_1–42_ appeared when CAP37 was incubated alone. The percent peak intensity for the full peptide gradually decreased and percent peak intensities for the fragment products increased with increasing incubation time in the presence of CAP37 (Fig 7c).

Incubation of Aβ_1–42_ with neutrophil elastase resulted in a rapid degradation of Aβ_1–42_. Immediately after the addition of neutrophil elastase (t≈0.25 min), the full Aβ_1–42_ peak was reduced to 28.83% (Fig 8c) and two fragment peaks were evident at 3616 Da and 3505 Da (Fig 8a). Five additional products (2877 Da, 2168 Da, 2069 Da, 1425 Da, and 1355 Da) predicted to be degraded Aβ_1–42_ were observed after incubation with neutrophil elastase for 60 min (Fig 8b). No peaks corresponding to the molecular mass of Aβ_1–42_ or the products of Aβ_1–42_ appeared when neutrophil elastase was incubated alone. The percent peak intensity of Aβ_1–42_ decreased from 28.83% to <1% by t = 5 min (Fig 8c). Percent peak intensities for the largest products that were generated (3616 Da and 3505 Da) decreased with increasing incubation time. For medium sized products (2877 Da, 2168 Da, and 2069 Da), percent peak intensities first increased at early time points, and then decreased with longer incubation time. The percent peak intensities for the smallest products observed (1425 Da and 1355 Da) appeared at t = 5 min incubation time with neutrophil elastase and gradually increased with increasing incubation time.

Aβ_1–42_ that was incubated with cathepsin G was also rapidly degraded. Immediately after adding cathepsin G (t≈0.25 min), the full-length Aβ_1–42_ peak represented only 27.43% of the total Aβ_1–42_ (Fig 9c) and three additional peaks predicted to be fragments of Aβ_1–42_ were present (Fig 9a, 2072 Da, 2001 Da, 1844 Da). The large peak appearing at 2464 Da was identified by MALDI TOF MS/MS as cathepsin G (data not shown). An additional fragment at 1476 Da was observed after Aβ_1–42_ incubation with cathepsin G for 5 min (Fig 9b and 9c). No peaks corresponding to the mass of Aβ_1–42_ or the products of Aβ_1–42_ appeared when cathepsin G was incubated alone. Aβ_1–42_ was completely degraded by t = 5 min (Fig 9c). The percent peak intensity for the largest product (2072 Da) gradually decreased with increasing incubation time. For medium sized fragments (2001 Da and 1844 Da), percent peak intensities first increased with early incubation time, and then decreased with longer incubation time. The percent peak intensity for the smallest product observed (1476 Da) appeared at t = 5 min incubation with cathepsin G and gradually increased with increasing incubation time.

**Fig 10:**
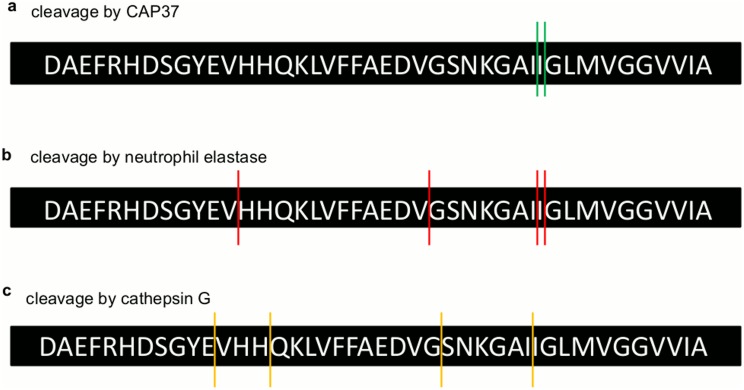
Sites of Aβ_1–42_ cleaved by CAP37, neutrophil elastase, and cathepsin G. Figure shows the sequence of Aβ_1–42_ and the sites at which Aβ_1–42_ is cleaved by (a) CAP37 (green lines), (b) neutrophil elastase (red lines), and (c) cathepsin G (yellow lines).

MALDI TOF MS/MS was used to determine the sequences of the resultant fragment peaks generated by CAP37, neutrophil elastase, and cathepsin G. Products at 3616 Da and 3505 Da that were generated by CAP37 were identified as Aβ_1–32_ and Aβ_1–31_ (Table 4), with cleavage sites between Ile^31^ and Ile^32^ and between Ile^32^and Gly^33^ (Fig 10a). CAP37, therefore, cleaved Aβ_1–42_ within its C-terminal region. Aβ_1–42_ products at 3616 Da and 3505 Da that were generated by neutrophil elastase were the same as those generated by CAP37 (Table 4). Therefore, Aβ_1–42_ was also cleaved at Ile^31^-Ile^32^ and Ile^32^-Gly^33^ by neutrophil elastase (Fig 10b). Products at 2877 Da and 1425 Da cleaved by neutrophil elastase were identified as Aβ_1–24_ and Aβ_1–12_. We were unable to identify the product at 2168 Da with MS/MS, and we have predicted putative sequences for 2069 Da and 1355 Da. The products at 2069 Da could be either Aβ_5–21_ or Aβ_21–42_ and product 7 could be either Aβ_11–21_ or Aβ_12–22_. Based on sequences confirmed with MS/MS, we demonstrated that neutrophil elastase also cleaved Aβ_1–42_ at Val^12^-His^13^ and Val^24^-Gly^25^ (Fig 10b). The fragments generated by cathepsin G were different from those generated by CAP37 and neutrophil elastase. Products at 2072 Da and 2001 Da were not identified with MS/MS, but putative sequences were predicted for each. The product at 2072 Da could be either Aβ_5–21_ or Aβ_21–42_ and product 2 could be either Aβ_21–41_ or Aβ_22–42_. With MS/MS, we identified products 3 and 4 as Aβ_15–31_ and Aβ_12–26_ (Table 4), respectively. Therefore, cathepsin G cleaved Aβ_1–42_ at Glu^11^-Val^12^, His^14^-Gln^15^, Gly^25^-Ser^26^, and Ile^31^-Ile^32^ (Fig 10c).

**Table 4:** Cleaved Aβ_1–42_ products generated by CAP37, neutrophil elastase, and cathepsin G.

Cleaved Aβ_1–42_ products			
**Generated by CAP37**			
*#*	*m/z*	*sequences confirmed by MS/MS*	*product*
1	3616	DAEFRHDSGYEVHHQKLVFFAEDVGSNKGAII	Aβ_1–32_
2	3505	DAEFRHDSGYEVHHQKLVFFAEDVGSNKGAI	Aβ_1–31_
**Generated by neutrophil elastase**			
*#*	*m/z*	*sequences confirmed by MS/MS*	*product*
1	3616	DAEFRHDSGYEVHHQKLVFFAEDVGSNKGAII	Aβ_1–32_
2	3505	DAEFRHDSGYEVHHQKLVFFAEDVGSNKGAI	Aβ_1–31_
3	2877	DAEFRHDSGYEVHHQKLVFFAEDV(G)	Aβ_1–24_
6	1425	DAEFRHDSGYEV(H)	Aβ_1–12_
		*Putative sequences*	
4	2168	Unknown	X
5	2069	(F)RHDSGYEVHHQKLVFFA(E) or (F)AEDVGSNKGAIIGLMVGGVIA	Aβ_5–21_ or Aβ_21–42_
7	1355	(Y)EVHHQKLVFFA(E) or (E)VHHQKLVFFAE(D)	Aβ_11–21_ or Aβ_12–22_
**Generated by cathepsin G**			
*#*	*m/z*	*sequences confirmed by MS/MS*	*product*
3	1844	QKLVFFAEDVGSNKGAI	Aβ_15–31_
4	1476	VHHQKLVFFAEDVG	Aβ_12–26_
		*Putative sequences*	
1	2072	(F)RHDSGYEVHHQKLVFFA(E) or (F)AEDVGSNKGAIIGLMVGGVIA	Aβ_5–21_ or Aβ_21–42_
2	2001	(F)AEDVGSNKGAIIGLMVGGVI(A) or (A)EDVGSNKGAIIGLMVGGVVIA	Aβ_21–41_ or Aβ_22–42_

## Discussion

The current study reveals a novel activity for neutrophil granule proteins CAP37, neutrophil elastase, and cathepsin G. Our data, describe for the first time that these three proteins are capable of disrupting the Aβ_1–42_ -RAGE interaction *in vitro*. Each of the three neutrophil proteins demonstrated distinct affinity and specificity for interacting with either RAGE or Aβ_1–42_. To our surprise, each of the neutrophil proteins also cleaved Aβ_1–42_. The neutrophil proteins disrupted the Aβ_1–42_ -RAGE interaction by competing with Aβ_1–42_ for the same binding site on RAGE, by sequestering Aβ_1–42_ away from RAGE, and/or by degrading Aβ_1–42_. Disruption of the Aβ_1–42_ -RAGE interaction by CAP37 appeared to be primarily due to its sequestering of Aβ_1–42_. Although we show that there is competitive inhibition by CAP37 for Aβ_1–42_ binding RAGE, the high ratio of CAP37: Aβ_1–42_ required for this inhibition makes it un-likely to occur *in vivo*. Since CAP37 cleaved Aβ_1–42_ at a very slow rate, it is also unlikely that CAP37 would disrupt the Aβ_1–42_ –RAGE by cleaving Aβ_1–42_. Disruption by neutrophil elastase was primarily due to proteolytic degradation of Aβ_1–42_ and was not a result of displacement of Aβ_1–42_ from RAGE since no competitive inhibition occurred in the presence of protease inhibitors. It is also unlikely that neutrophil elastase would sequester Aβ_1–42_ since the affinity of Aβ_1–42_ for neutrophil elastase is low. Cathepsin G bound to both RAGE and Aβ_1–42_, and also rapidly degraded Aβ_1–42_. The fact that cathepsin G bound to RAGE, but binding was not significantly reduced with the RAGE antagonist, GM-0111, suggests that this interaction may be due to high electrostatic potential and/or that cathepsin G binds to a non-canonical site on RAGE that is not blocked by the GM-0111 antagonist.

We presume that neutrophil elastase and cathepsin G degraded Aβ_1–42_ due to their serine protease activities. Neutrophil elastase and cathepsin G are classified as serine proteases since they depend on a serine residue for their catalytic activity [31]. Serine proteases have been further classified based on their evolutionary origins. Neutrophil elastase and cathepsin G belong to the PA clan, based on their 3D structure (double β barrel fold) and catalytic triad formed by Histidine (His), Asparagine (Asp), and Serine (Ser) residues [31, 32]. Substrates cleaved by neutrophil elastase and cathepsin G include proteins involved in maintaining the integrity of the extracellular matrix such as fibronectin, laminin, and proteoglycans [33–35]. Neutrophil elastase also cleaves elastin and collagens [35]. Cathepsin G cleaves various proteins involved in blood clotting [36]. In addition, both neutrophil elastase and cathepsin G cleave important immune regulators including complement component 3 [37], complement component 5a receptor (C5aR) [38], and stromal cell-derived factor-1 alpha (SDF-1α) [39, 40]. All six insulin-like growth factor binding proteins, which mediate insulin-like growth factor signaling, are also cleaved by neutrophil elastase and cathepsin G [41]. An important consideration is that even though an increase in neutrophil elastase or cathepsin G was not observed in the brains of AD patients in our previous report [10], their enzymatic activities were not determined and may be different in AD patients. Although CAP37 has the same 3D structure as neutrophil elastase and cathepsin G, it has been thought to be an inactive serine protease due to the absence of the conserved His-Asp-Ser catalytic triad replaced by Ser-Asp-Gly [42, 43]. However, recent studies have shown that CAP37 cleaves insulin-like growth factor binding proteins-1,-2, and -4 [44, 45]. Since we demonstrated that CAP37 cleaves Aβ within the C-terminal region, the current study upholds these previous findings indicating that CAP37 does have proteolytic activity. Whether there could be an increase or decrease in CAP37 activity in patients with AD will also need to be determined.

Although other serine proteases including plasmin, myelin basic protein, and acylpeptide hydrolase have been revealed as amyloid beta degrading proteases (AβDPs) [46], this is the first report of neutrophil serine proteases degrading Aβ_1–42_. A previous study demonstrated the ability of cathepsin G to cleave amyloid precursor protein (APP) before the N-terminus of the Aβ_1–42_ peptide [47]. However, degradation of the Aβ_1–42_ peptide itself was not explored. The exact cleavage sites and the kinetics of cleavage were also not investigated in this report. One of the most well-known AβDPs, neprilysin, was found to be identical to skin fibroblast elastase. While both neprilysin and neutrophil elastase have been shown to hydrolyze the premature elastic fibers, oxytalan and elaunin [48], the two enzymes are otherwise distinguishable. Unlike neutrophil elastase, neprilysin is a metalloprotease, and cleaves various peptide hormones including substance P and Bradykinin [49, 50].

The exact sequence of each Aβ_1–42_ fragment formed by cleavage catalyzed by each of the neutrophil proteins is still uncertain, but it is clear that large fragment products are formed first, followed by intermediate sized products, and the smallest products are produced last. Each of the three neutrophil proteins demonstrated distinct kinetics for degrading Aβ_1–42_, and cleaved at different sites within the peptide. All three proteins did cleave at Ile^31^-Ile^32^, which was likely one of the initial cleavage sites since the corresponding Aβ_1–31_ is one of the largest products and was formed immediately after Aβ_1–42_ incubation with neutrophil elastase. Cleavage at Val and Gly residues by neutrophil elastase and at Glu, Gln, and His residues by cathepsin G are typical sites of cleavage for these two enzymes [31, 51]. Although we observed Aβ_1–31_ and Aβ_1–32_ products after incubation with CAP37 or neutrophil elastase, we did not observe these products after incubation with cathepsin G. However, it seems possible that these products and others may have been immediately formed and further degraded to form Aβ_15–31_ and Aβ_12–26_ upon incubation with cathepsin G. Since Aβ_12–26_ could not be a cleaved product derived from Aβ_15–31_ or vice versa, it is probable that the two fragments derived from two or more other fragments and were degraded before our analysis. It is uncertain why cathepsin G was truncated to produce a fragment of ~2464 Da only in the presence of Aβ_1–42_ and not when incubated alone. The strong binding of Aβ_1–42_ to cathepsin G could have promoted allosteric regulation of cathepsin G by Aβ_1–42_ causing cathepsin G to be more vulnerable to self-cleavage. It is interesting that CAP37 only cleaved Aβ_1–42_ at Ile residues, because cleavage of insulin-like growth factor binding protein-1 by CAP37 was also found to occur at an Ile residue [44]. The substrate specificity of CAP37 is currently unknown, but based on these findings, it may be possible that CAP37 has a specificity towards hydrophobic aliphatic amino acids. Whether or not the modified catalytic triad of CAP37 allows for the catalytic activity is unknown. Further studies will be needed to determine the exact mechanism of action.

In a study by Chaney et al., [52] molecular modeling was used to predict that RAGE interacted with the N-terminal domain of Aβ. Our analysis indicates that the N-terminus of Aβ_1–42_ is left intact in the presence of CAP37 but is cleaved by both neutrophil elastase and cathepsin G. This could explain why protease inhibitors prevented neutrophil elastase, but not CAP37, from disrupting binding of Aβ_1–42_ to RAGE (Fig 4). Smaller fragments of Aβ_1–42_ have been demonstrated to be less toxic than the full peptide in cultured brain endothelial cells and SH-SY5Y neuroblastoma cells [53]. Even Aβ_1–40_ is less toxic than Aβ_1–42_ and does not form toxic aggregates as readily [54–56]. Therefore, it is possible that each of the neutrophil proteins could modulate the neurotoxicity of Aβ_1–42_ by degrading it and/or by reducing its interaction with RAGE. Further studies using cell culture and animal models should be conducted to determine the functional outcomes of these neutrophil protein activities.

Only recently have reports began to unveil neutrophils as factors involved in the progression of AD. One study revealed increased numbers of neutrophils in patients with AD as well as in two different transgenic mouse models of AD [57]. Interestingly, depletion of neutrophils was able to improve memory in transgenic AD mice and reduce pathology, including Aβ load and microgliosis. Another report demonstrated that neutrophils migrated towards Aβ plaques in a mouse model of AD but not in wild-type mice [58]. Our findings that neutrophil proteins are capable of binding and degrading Aβ_1–42_ makes it tempting to speculate whether neutrophils may be migrating toward plaques in an attempt to promote Aβ clearance.

CAP37 may not only be released from neutrophils to interact with Aβ, but may also interact with Aβ inside neurons. Aβ has been reported to accumulate inside neurons and promote dysfunction of organelles [59, 60]. The fact that we previously identified CAP37 expression in neurons signifies a potential interaction between Aβ and CAP37 within neurons. Additional studies are needed to determine if CAP37 could cleave Aβ in neurons or modulate its effects on intracellular organelles. Although neutrophil elastase and cathepsin G have been detected in mouse microglial cells, it is uncertain whether these proteins are expressed within neurons and if they could interact with intraneuronal Aβ.

Possible clearance of Aβ by neutrophils in the periphery could also have important implications. Previous reports have indicated that peripheral clearance of Aβ may reduce Aβ accumulation in the brain [61, 62]. In a recent study by Xiang et al., [63] a mouse model of parabiosis was used in which the bloodstreams of APP transgenic mice were connected to the bloodstreams of wild-type mice. Interestingly, parabiosis significantly reduced blood levels of Aβ as well as brain Aβ plaque burden in the APP transgenic mice compared to APP mice which did not undergo parabiosis. This report indicates that peripheral catabolism of Aβ may be sufficient to prevent its accumulation in the brain. Therapies to effectively promote this catabolism still need to be developed. Neutrophil proteins such as CAP37, neutrophil elastase, and cathepsin G that can sequester or degrade Aβ might support this catabolism.

**Fig 11:**
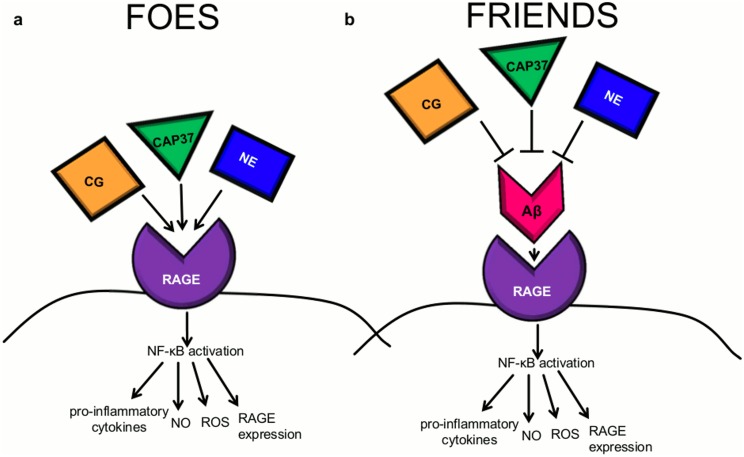
Proposed model for the modulation of RAGE signaling by CAP37, neutrophil elastase, and cathepsin G. (a) CAP37, neutrophil elastase (NE), and/or cathepsin G (CG) may act as RAGE agonists to induce a cell signaling cascade that leads to activation of the transcription factor NF-κB. Activation of NF-κB could then stimulate the production of pro-inflammatory cytokines and pro-oxidants including reactive oxygen species (ROS) and nitric oxide (NO) as well as RAGE itself. In this way, the neutrophil proteins would likely act as foes in the course of chronic inflammatory disease such as AD. (b) CAP37, neutrophil elastase, and cathepsin G may prevent the Aβ_1-42_-RAGE interaction and the corresponding NF-κB signaling cascade. In this way, the neutrophil proteins would act as friends to prevent chronic neuroinflammation driven by Aβ_1–42_ activation of RAGE.

The current study indicates that CAP37, neutrophil elastase, and cathepsin G may be neuroprotective in the course of AD by decreasing the Aβ-RAGE interaction. However, by binding to RAGE, the neutrophil proteins may also be able to act as RAGE agonists to elicit signaling through RAGE independently of their effects on Aβ_1–42_. If this is the case, the neutrophil proteins could act as both friends and foes in the course of chronic neuroinflammatory diseases such as AD (see Fig 11 for hypothetical model).

## Conclusions and Future Directions

We conclude that the neutrophil proteins CAP37, neutrophil elastase, and cathepsin G could play an important role in regulating the Aβ_1–42_ -RAGE interaction in cells of the brain and/or periphery. Disrupting this interaction could be neuroprotective in diseases such as AD, in which Aβ_1–42_ -RAGE interactions may contribute to the chronic inflammation, oxidative stress, and brain Aβ accumulation associated with disease pathology. Further studies must be conducted in cell culture and *in vivo* to determine how these proteins could affect neurotoxicity by mediating the Aβ_1–42_ -RAGE interaction. GM-0111 can be utilized in these future studies to determine if the observed effects of neutrophil proteins in cell culture/*in vivo* are RAGE-dependent. Since the GM-0111 not only acts as a RAGE antagonist, but also inhibits the activity of neutrophil elastase, it could also be incorporated into future studies aimed to determine if neutrophil elastase prevents neurotoxicity through its enzymatic activity against Aβ_1–42_.
